# Isolation and Characterization of *Faecalibacterium prausnitzii* from Calves and Piglets

**DOI:** 10.1371/journal.pone.0116465

**Published:** 2014-12-31

**Authors:** Carla Foditsch, Thiago M. A. Santos, Andre G. V. Teixeira, Richard V. V. Pereira, Juliana M. Dias, Natália Gaeta, Rodrigo C. Bicalho

**Affiliations:** 1 Department of Population Medicine and Diagnostic Sciences, College of Veterinary Medicine, Cornell University, Ithaca, New York, United States of America; 2 Department of Biochemistry, University of Wisconsin–Madison, Madison, Wisconsin, United States of America; National Institute of Agronomic Research, France

## Abstract

The goal of our study was to isolate and characterize *Faecalibacterium prausnitzii* from fecal samples of healthy calves and piglets, in order to develop a novel probiotic for livestock animals. We identified 203 isolates of *Faecalibacterium sp.*, which were clustered in 40 genetically distinct groups. One representative isolate from each cluster was selected for further characterization. The concentrations of the short chain fatty acids (SCFA) acetate, butyrate, propionate and isobutyrate in the culture media were measured by gas chromatography. We observed reduction in the concentration of acetate followed by concomitant increase in the concentration of butyrate, suggesting that the isolates were consuming acetate present in the media and producing butyrate. Butyrate production correlated positively with bacterial growth. Since butyrate has many benefits to the colonic epithelial cells, the selection of strains that produce higher amounts of butyrate is extremely important for the development of this potential probiotic. The effect of pH and concentration of bile salts on bacterial growth was also evaluated in order to mimic the conditions encountered by *F. prausnitzii in vivo*. The optimal pH for growth ranged between 5.5 and 6.7, while most isolates were inhibited by of the lowest concentration of bile salts tested (0.1%). Antimicrobial resistance profile showed that most isolates of *Faecalibacterium sp.* were resistant against ciprofloxacin and sulfamethoxazole-trimethoprim. More than 50% of the isolates were resistant to tetracycline, amikacin, cefepime and cefoxitin. A total of 19 different combinations of multidrug resistance were observed among the isolates. Our results provide new insights into the cultural and physiological characteristics of *Faecalibacterium prausnitzii* illustrating large variability in short chain fatty acid production, in vitro growth, sensitivity to bile salts, and antibiotic resistance and suggesting that future probiotic candidates should be carefully studied before elected for *in vivo* studies.

## Introduction

The gut microbiota plays a key role in shaping various aspects of postnatal life; it contributes to the development of the immune system [1], [2] and controls energy balance by influencing energy expenditure and storage [3]. Higher ratio of *Firmicutes* to *Bacteroidetes* in the gut has been associated with obesity in mice [4]. Specifically, the level of *Faecalibacterium prausnitzii* has been shown to increase significantly in the microbiota isolated from fecal samples of obese children when compared to the microbiota from non-obese individuals [5]. These findings suggest that *F. prausnitzii* is related to the energy harvesting capacity of the intestinal microbiota.

Low levels of *F. prausnitzii* have been associated with chronic inflammatory disorders of the gastrointestinal tract, including Crohn's disease [6] and ulcerative colitis [7]. Recent research has shown that *F. prausnitzii* has anti-inflammatory and immunomodulatory capacities, which improved the 2, 4, 6-trinitrobenzenesulfonic acid (TNBS)-induced colitis in mice, partially due to secreted metabolites blocking NF-κB activation and IL-8 production [8], [9]. Therefore, the use of *F. prausnitzii* as a probiotic might be a promising strategy for the treatment of Crohn's disease.

*F. prausnitzii* (formally *Fusobacterium prausnitzii*) is a Gram-positive, non-motile, non-spore-forming, butyrate-producer bacterium belonging to the *Firmicutes* phylum [10]. It is part of the normal intestinal microbiota of many animal species and represents one of the most abundant bacteria encountered in the feces of healthy animals, such as humans [11]–[13], bovine [14], swine [15], mice [16], and poultry [17].

*F. prausnitzii* is a major butyrate producer [18]. Butyrate produces the highest energy value per mole compared to other short chain fatty acids (SCFA) [19], it is an important energy source to the colonic epithelial cells, it has anti-inflammatory and epithelial barrier-preserving effects, and it regulates cell proliferation, differentiation, and apoptosis [20]. Butyrate has been proven to be beneficial to livestock as well; higher levels of butyrate in the rumen of growing steers were associated with better feed conversion efficiency [21]. A positive effect on body weight gain, health, and metabolic intermediates (plasma glucose, serum total protein, plasma glucagon-like peptide-2) was observed when sodium butyrate was supplemented to calves in the milk replacer or starter mixture [22]. Additionally, the same study showed that it indirectly stimulated the rumen development. We previously characterized the fecal microbiota of calves during the pre-weaning period using a metagenomic approach and provided evidence of the potential beneficial effects of *F. prausnitzii* in the intestinal tract of neonatal calves [14]. Higher prevalence of *Faecalibacterium* sp. in the first week of life of Holstein calves was associated with improved weight gain and decrease in the incidence of diarrhea. The role of *F. prausnitzii* in energy harvesting and its anti-inflammatory effects might explain the results observed in the performance of the calves. Here, we characterize *Faecalibacterium prausnitzii* isolated from feces of calves and piglets to study cultural and physiological aspects of this commensal bacterium and to substantiate its use as a viable animal probiotic.

## Materials and Methods

### Ethics statement

Fecal samples were collected calves that were housed on a large commercial dairy farm located near Ithaca NY and from piglets housed on Cornell University facilities. The research protocol was reviewed and approved by the Institutional Animal Care and Use Committee of Cornell University (Protocol number: 2012–0055). The fecal sample collections from the calves housed on a commercial dairy farm was authorized by the farm owner, who was aware of the procedure.

### Bacterial strains and growth conditions

A reference strain of *F. prausnitzii* used as a control in our experiments was obtained from the DSMZ-German Collection of Microorganism and Cell Cultures (DSM17677, strain designation A2-165) [10], [18]. We developed a complex culture medium (herein referred to as VTR2RF) containing rumen fluid. The VTR2RF media used as transport, enrichment, and isolation media was composed of the anaerobic media Versa TREK REDOX 2 (Trek Diagnostic Systems, Cleveland-OH) supplemented with 30% filtered rumen fluid. Rumen fluid was collected from fistulated cows, centrifuged at 12,000×*g* for 30 min, the supernatant was filter-sterilized (Corning Incorporated Life Sciences, Tewksbury-MA) 3 times and stored at 4°C. VTR2RF agar was additionally supplemented with 0.5% (w/v) yeast extract (BD, Franklin Lakes, NJ), 5 mg/l (w/v) hemin (Sigma-Aldrich, St. Louis, MO), 1 mg/l (w/v) cellobiose (Sigma-Aldrich), 1 mg/ml (w/v) maltose (Sigma-Aldrich), and 0.5 mg/ml (w/v) L-cystein (Sigma-Aldrich) [9].

### Sample collection and isolation of anaerobic bacteria

Fecal samples were collected from 7–28 days-old healthy Holstein calves and from 10–30 days-old healthy piglets. The samples were gently collected from the rectum and immediately placed in a tube containing 12 ml of VTR2RF broth. The tubes were sealed and transported until further processing. The subsequent procedures were performed in an anaerobic chamber (BacBasic chamber, Sheldon Manufacturing, Inc., Cornellius, OR). All samples were serially diluted in Anaerobic Dilution Blank (Anaerobe Systems, USA) and plated on VTR2RF agar. After 48 h, about 10 typical colonies from each sample were selected and single-colony purified in VTR2RF agar. The isolates were stored at −80°C in VTR2RF broth containing 16% of glycerol.

### DNA extraction, PCR and 16S rDNA sequencing

Genomic DNA was extracted from typical colonies using the InstaGene Matrix (Biorad, Hercules, CA) according to the manufacturer's instructions with some modifications. Briefly, one colony was inoculated in 200 µl of InstaGene Matrix, incubated at 56°C for 30 min, mixed using a vortex and incubated at 100°C for 8 min. After centrifugation at 13,400×*g* for 1 min, the supernatant was collected and used for the PCR.

The DNA concentration was measured using a spectrophotometer (Nanodrop^TM^ 1000 – NanoDrop Technologies, Rockland, DE) and, approximately, 350 ng of genomic DNA were used for PCR. The 16S rDNA gene was amplified by PCR using the universal eubacterial primers fD1 (forward primer) and rP2 (reverse primer) [23]. The 100-µl reaction mix was composed of 50 µl of Green GoTaq Master Mix (2× Green GoTaq Master consisting of Green GoTaq Reaction Buffer, 400 µM of each dNTP, and 3 mM MgCl_2_; Promega, Madison, WI), 20 pmol of each primer, 350 ng of DNA, and nuclease-free water. Reaction conditions for the amplification were an initial cycle of 94°C for 5 min, 57°C for 2 min, and 72°C for 2 min, followed by 29 cycles of 94°C for 2 min, 57°C for 30 sec, and 72°C for 2 min, with a final cycle of 72°C for 10 min [23].

The PCR products were purified using the QIAquick PCR Purification Kit (Qiagen, Germantown, MD), according to the manufacturer's protocol, and sequenced at the Cornell University Life Sciences Core Laboratories Center. The sequences obtained were compared to sequences deposited in the Ribossomal Database Project Classifier (RDP – Center for Microbial Ecology, Michigan State University, East Lansing MI).

### RAPD analysis

The genetic diversity of the isolates was assessed by random amplified polymorphic DNA PCR (RAPD-PCR) using the primer 1254 [24]. Amplification products were resolved by electrophoresis in a 2% (w/v) agarose gel and stained with 0.5 µg/ml of ethidium bromide [25]. The RAPD-PCR profiles were visually compared and clustered in 40 genetically distinct groups of isolates. One representative isolate from each cluster was selected for further characterization, as described below.

### Growth performance

The inoculum cultures were grown under the same conditions and standardized by optical density. Half microliter of each inoculum were inoculated in 40 ml of VTR2RF broth and incubated at 37°C for 48 h under anaerobic conditions. Two aliquots were aseptically removed from each bottle and the optical density (O.D.) of the culture sample was measured at λ = 600 nm in a Synergy 2 Microplate Reader (BioTek Instruments, Inc., Winooski, VT). The difference in average O.D. between 5 h and 48 h of incubation was used to calculate the growth performance. The procedure was repeated, at least, 2 times for each sample.

### Short chain fatty acids (SCFA) metabolism

*Faecalibacterium sp.* isolates and the reference strain DSM 17677 were inoculated in 25 ml of VTR2RF broth and incubated at 37°C for 48 h under anaerobic conditions. The culture was centrifuged at 4000×*g* for 10 min and the supernatant was collected. The concentration of acetate, butyrate, propionate and isobutyrate in the VTR2RF media before inoculation and in the supernatant of the culture was measured by gas chromatography. Samples were injected into a Perkin Elmer Autosystem XL Gas Chromatograph containing a Supelco packed column (Sigma-Aldrich, St. Louis, MO) and the analysis was performed according to the manufacturer's protocol [26] at the Dairy One Cooperative, Ithaca, NY. The procedure was repeated, at least, 3 times for each sample.

### Resistance to pH and bile salts

The pH of VTR2RF broth was adjusted to 6.7, 6.2, 5.5, 5.0, 4.5, 4.0 and 3.5 with HCl and the adjusted broth was inoculated with the *Faecalibacterium sp.* isolates or the reference strain DSM 17677. The O.D. of the cultures was measured at λ = 600 nm in a Synergy 2 Microplate Reader (BioTek Instruments, Inc.) 0 h and 48 h after inoculation. Similarly, we evaluated bacterial growth in VTR2RF broth supplemented with 0.1% (wt/vol), 0.25% (wt/vol), and 0.5% (wt/vol) bile salts (Sigma-Aldrich, St. Louis, MO) [24].

### Disk diffusion and Etest MIC

A preliminary susceptibility test was performed using the disk diffusion agar assay, adapted from the Performance Standards for Antimicrobial Disk Susceptibility Tests (CLSI, 2012). To standardize the inoculum density, the direct cell suspension was adjusted according to the 0.5 McFarland turbidity standard. The suspension was spread with a swab on a 150-mm VTR2RF agar plate and 12 antibiotic disks (ampicillin, cefoxitin, ceftiofur, ceftriaxone, chloramphenicol, ciprofloxacin, enrofloxacin, nalidixic acid, neomycin, streptomycin, sulfamethoxazole-trimethoprim and tetracycline) were deposited on the agar. The plates were also incubated at 37°C for 48 h under anaerobic conditions. The diameters of zones of complete inhibition were measured with calipers.

An additional determination of the minimal inhibitory concentration (MIC) was performed by Etest (BioMérieux, Inc., Durham, NC), which consist of plastic strips coated with antimicrobials that create a concentration gradient as they diffuse into the agar [27]. To standardize the inoculum density, the direct cell suspension was adjusted according to the 1.0 McFarland turbidity standard. The suspension was spread with a swab on two 150-mm VTR2RF agar plates and five Etest strips were added per plate, according to the manufacturer's instructions. The plates were incubated at 37°C for 48 h under anaerobic conditions. The intersection of the ellipse of growth inhibition with the strip was considered as the MIC. The antibiotics tested were amikacin, ampicillin, cefepime, cefoxitin, ceftriaxone, chloramphenicol, ciprofloxacin, gentamicin, tetracycline and sulfamethoxazole-trimethoprim. For both the disk diffusion assay and the Etest, quality assurance was performed using *Bacteroides fragilis* ATCC 25285, as recommended by the CLSI (CLSI, 2004).

We estimated the resistance profile of the *Faecalibacterium sp.* isolates by comparing the MIC values with the standard values determined by the CLSI for *Bacteroides fragilis* ATCC 25285 (CLSI, 2004) and reported elsewhere [28], [29]. No published MIC breakpoint for amikacin and gentamicin were found in the literature, therefore the highest concentrations were considered as the MIC for these two aminoglycosides.

### Phylogenetic analysis

The 16S rDNA sequences were aligned and manually trimmed using the cross-platform bioinformatics software *Geneious* 7.0.6 (Biomatters, Auckland, New Zealand). The sequences were compared with other sequences imported from the NCBI database. Sequences from bacteria belonging to other families of the *Clostridiales* order (*Ruminococcaceae, Syntrophomonadaceae, Peptostreptococcaceae, Clostridiaceae* and *Lachnospiraceae*) were included in the phylogenetic analysis. The sequences of our isolates have been deposited in GenBank (NCBI) under the accession numbers KJ957841 to KJ957877.

### Statistical Analyses

Data were analyzed using JMP Pro (version 11, SAS Institute Inc., Cary, NC). Results are presented as mean and standard error of the mean, and as correlation coefficient (r) matrix for the multivariate analysis between the average difference of SCFA concentrations and the growth performance. Box and whiskers plots were generated using MedCalc Statistical Software version 13.1.2 (MedCalc Software, Ostend, Belgium).

## Results

### *Faecalibacterium sp.* isolation and identification

A total of 931 anaerobic bacteria were isolated from fecal samples collected from approximately 130 healthy Holstein heifer calves and 6 isolates were obtained from samples collected from 10 piglets. Based on the 16S rDNA sequences, 203 of these isolates were identified as *Faecalibacterium* sp., which were clustered in 40 genetically distinct groups according to the RAPD-PCR profiles. One representative isolate from each cluster was used for the subsequent analysis.

### Growth performance

**Figure 1:**
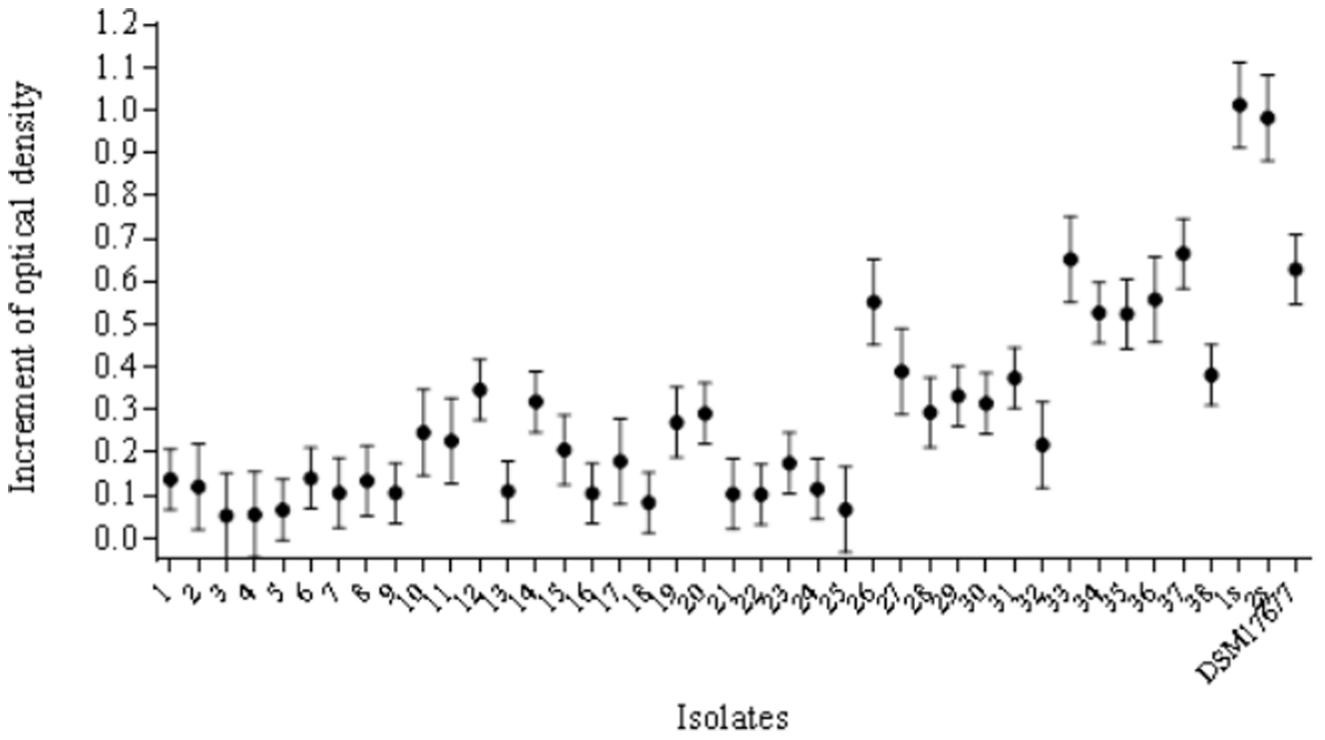
The growth of each of our 40 isolates and the DSM 17677 isolate, measured spectrophotometrically at hours 5, 17, 29, 41 and 48 using absorbance at the 600 nm wavelength. The average optical density increment was calculated as the difference between 5 h and 48 h of incubation. Error bars indicate the standard error of the mean.

An increment in O.D. was observed for all isolates after 48 h of incubation (Fig. 1). This increment was higher for the isolates that produced more butyrate suggesting that the growth rate is associated with higher butyrate production.

### Short chain fatty acids (SCFA) metabolism

**Figure 2:**
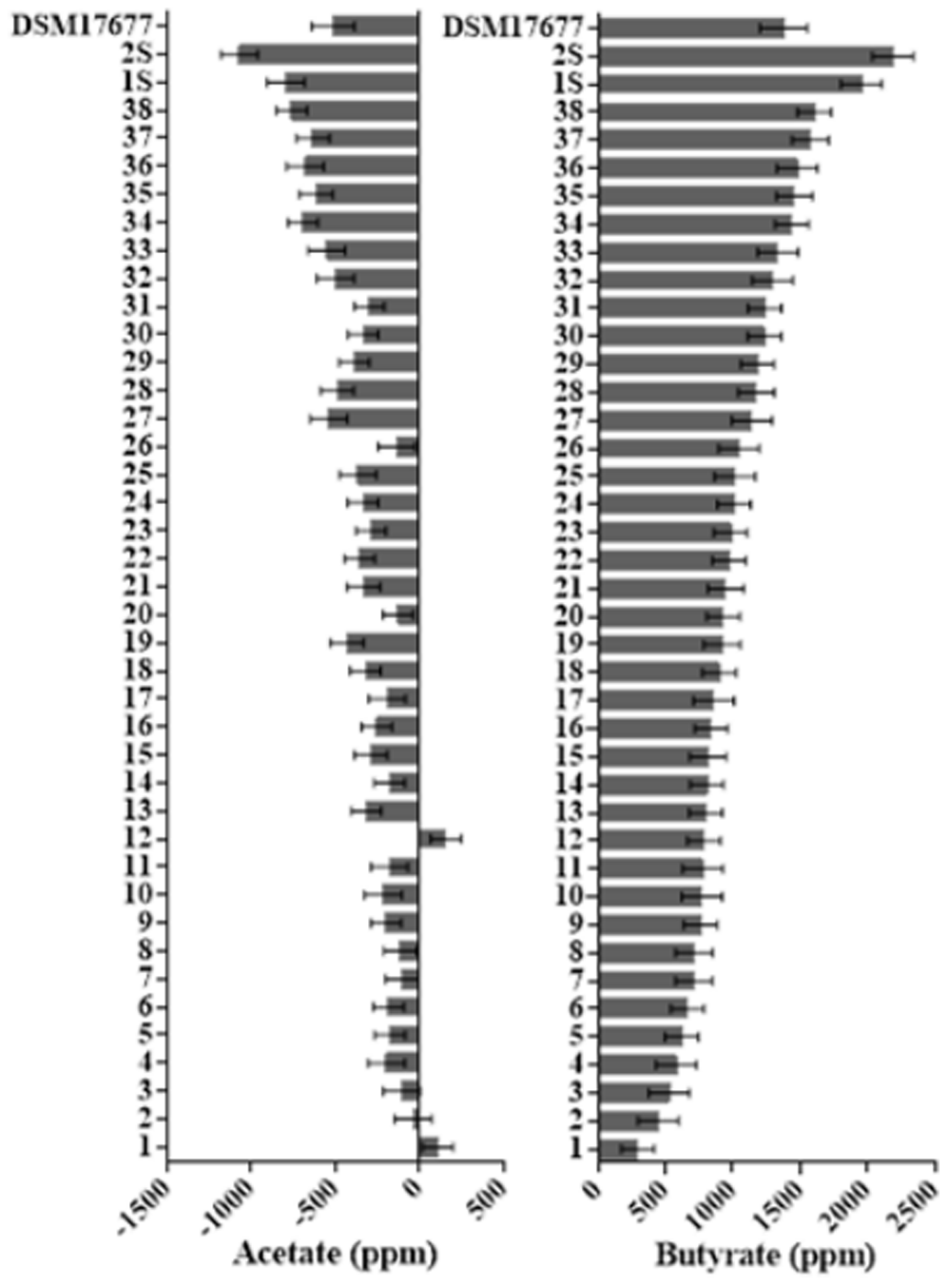
Average difference of acetate and butyrate concentrations in the growth media after 48 h of incubation of each *F. prausnitzii* isolate. The bars represent the mean concentration in ppm. Error bars indicate the standard error of the mean. Based on the butyrate production, the *F. prausnitzii* isolates were entitled from 1–38 (bovine), and 1S and 2S (swine).

**Figure 3:**
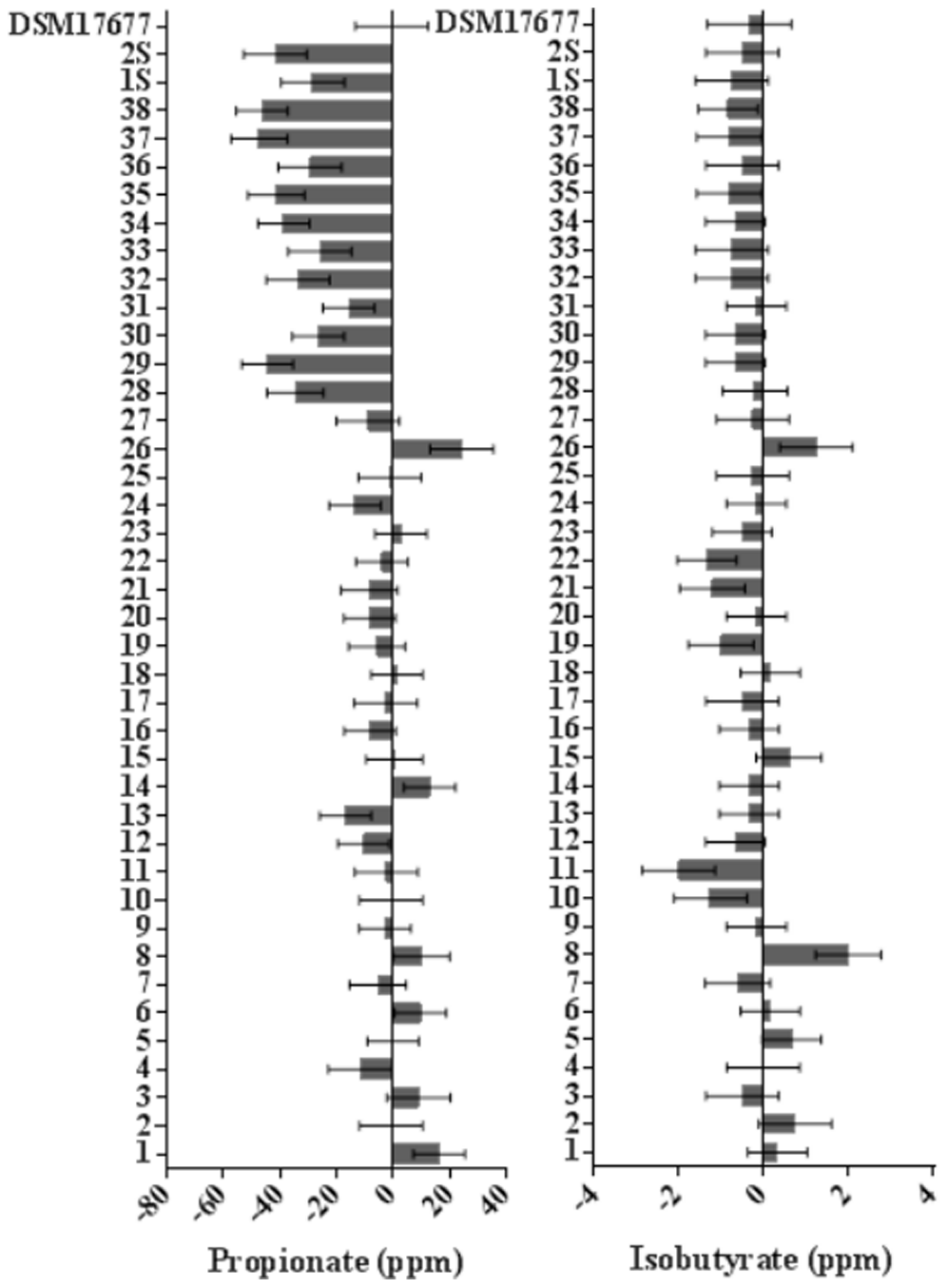
Average difference of propionate and isobutyrate concentrations in the growth media after 48 h of incubation of each *F. prausnitzii* isolate. The bars represent the mean concentration in ppm. Error bars indicate the standard error of the mean.

**Figure 4:**
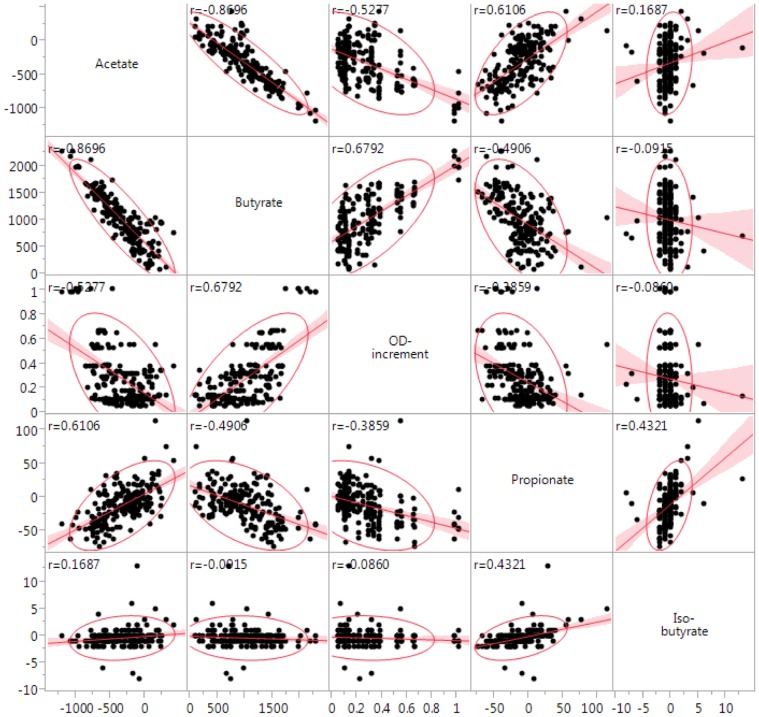
Correlations between the average difference of short chain fatty acids (acetate, butyrate, propionate and isobutyrate) concentrations and the growth performance of the *F. prausnitzii* isolates, represented by the increment of optical density. Correlation coefficients and fitted lines with confidence intervals are shown in each box.

The average difference of the acetate and butyrate concentrations in the growth media before and after 48 h of incubation for each *Faecalibacterium sp.* isolate is shown in Fig. 2. The identification (bovine 1–38, swine 1S, 2S) of each *Faecalibacterium* sp. isolate described here was based on the butyrate production. After 48 h of incubation, there was an evident reduction in acetate concentration and concomitant increase in butyrate concentration. The average differences of propionate and isobutyrate concentrations in the medium before and after 48 h of growth are shown in Fig. 3. The concentration of propionate and isobutyrate decreased for most of the isolates; however, the differences in concentrations were not as conspicuous as the difference detected for acetate and butyrate. The scatterplot matrix (Fig. 4) shows the correlations between the average difference of SCFA concentrations and the growth performance (fitted lines with confidence intervals are also shown). The correlation between the average difference of butyrate and the mean OD increment was r = 0.68. There was a high negative correlation (r = −0.87) between the acetate and butyrate average concentrations, meaning that the high butyrate producers were consuming more acetate as well.

### Resistance to different pH and bile salts

**Figure 5:**
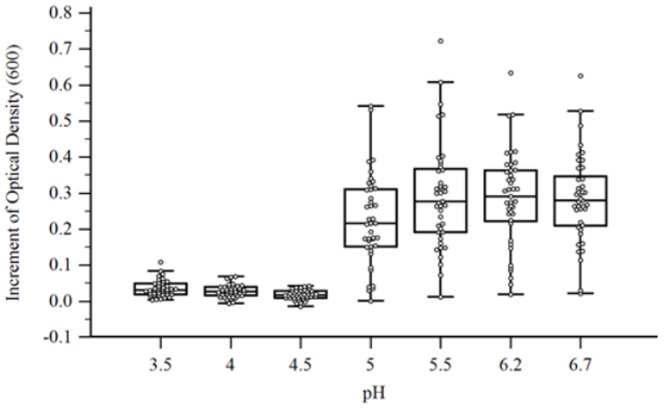
Effect of pH on bacterial growth. The increment of optical density after 48 h of incubation for each isolate in media containing different pH. Each box-and-whisker plot constitutes one pH value. The extent of the box encompasses the interquartile range of the optical density increment, whiskers extend to maximum and minimum values, and the line within each box represents the median. Outliers are represented as open black circles.

**Figure 6:**
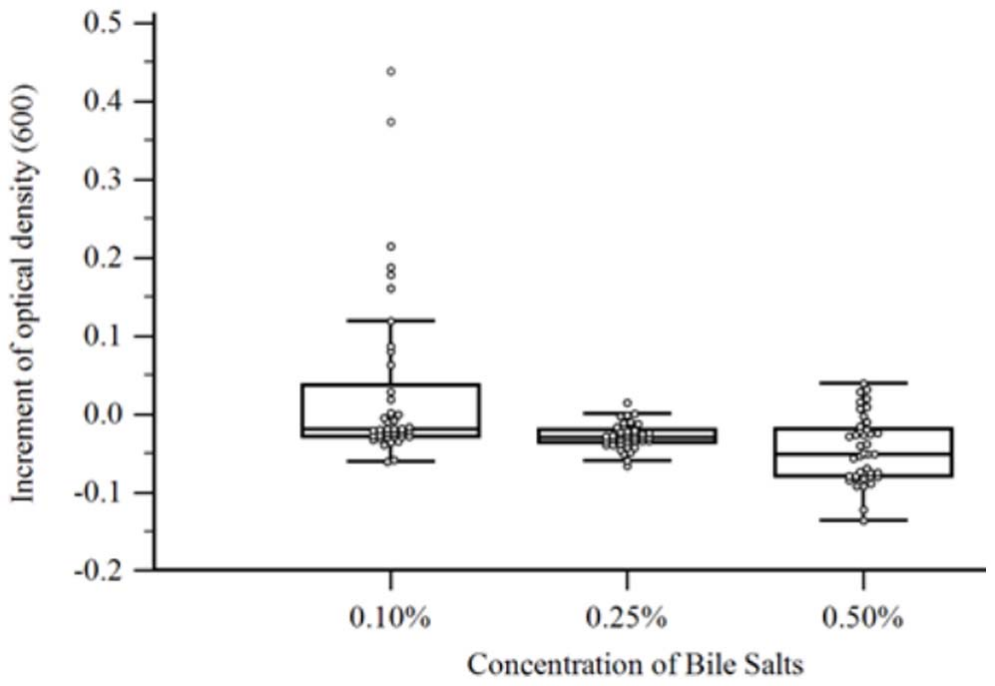
Effect of bile salts on bacterial growth. The increment of optical density after 48 h of incubation for each isolate in media containing different bile salts concentrations are displayed. Box-and-whisker plots represent each bile salt concentration (0.1, 0.25 and 0.5%).

Bacterial growth was observed in pH values between 5.0 and 6.7 (Fig. 5). No growth was observed in pH 4.5, 4.0, or 3.5. Bile salts inhibited growth of the majority of the isolates (Fig. 6) and only few isolates were able to grow in the presence of 0.1% (wt/vol) bile salt.

### Disk diffusion and Etest MIC

**Figure 7:**
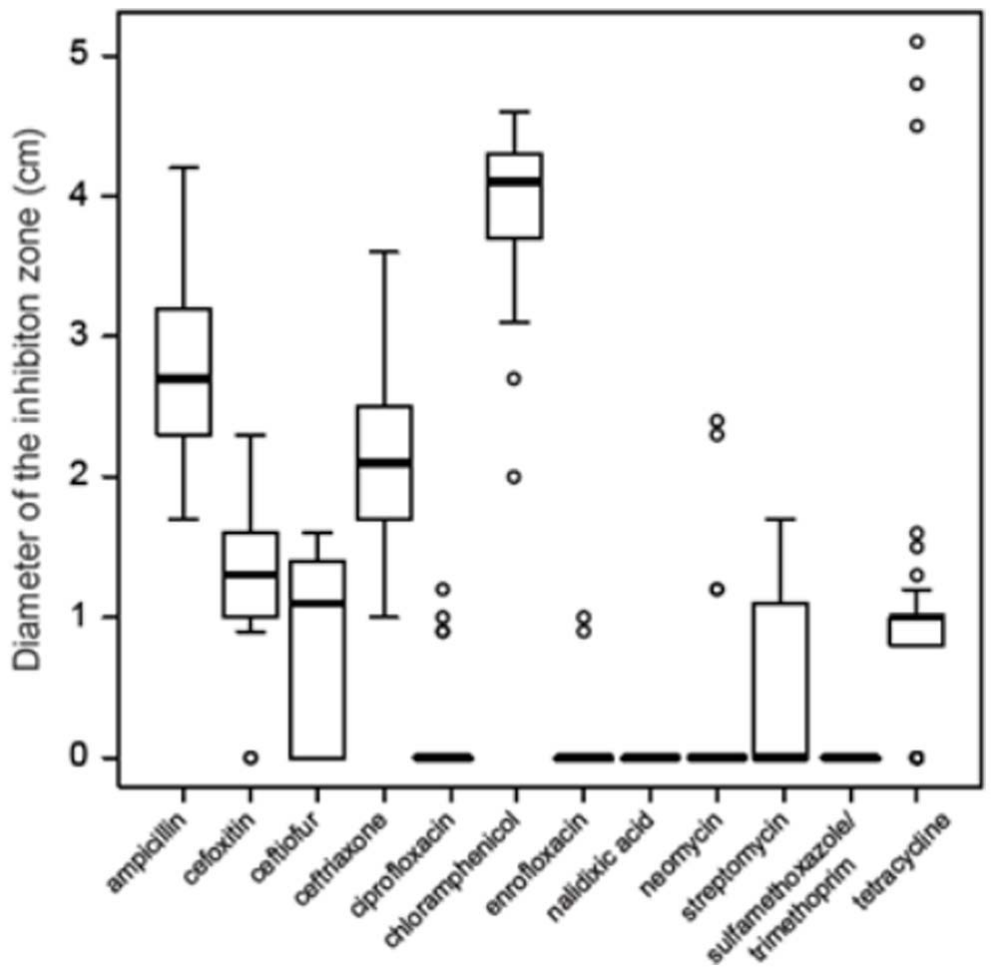
Box-and-whisker plots depicting the diameter of the inhibition zone measured for each antibimicrobial agent, as determined by the disk diffusion agar assay.

Ciprofloxacin, enrofloxacin, nalidixic acid, neomycin and sulfamethoxazole-trimethoprim disks did not inhibit the growth of most isolates (Fig. 7). Cefoxitin, ceftiofur and streptomycin had a small diameter of inhibition; the means and standard deviations were 1.33 cm 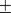 0.48, 0.85 cm 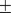 0.64, and 0.56 cm 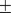 0.63, respectively. The bovine isolates had a small inhibition zone for tetracycline (0.76 cm 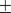 0.49), which was different from the observed for the swine isolates and the DSM 17677 that ranged from 4.5 to 5.1 cm. Larger inhibition zones were observed for most of the isolates in the presence of ampicillin (2.75 cm 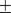 0.61), ceftriaxone (2.11 cm 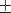 0.62) and chloramphenicol (3.98 cm 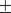 0.52).

According to the results obtained for the Etest (Table 1), all isolates were resistant to ciprofloxacin and sulfamethoxazole-trimethoprim; 82.8% were resistant to tetracycline; 55.2% were resistant to cefepime and cefoxitin; 34.5% were resistant to ceftriaxone; 27.6% were resistant to ampicillin; and none of the isolates was resistant to chloramphenicol. About 55.2% of the isolates grew in the highest concentration of amikacin tested and only 1 isolate was resistant to gentamicin.

**Table 1:** Resistance profile of the *Faecalibacterium prausnitzii* isolated from feces of dairy calves and piglets, as determined by E-test (BioMérieux, Inc., Durham, NC).

	Distribution of isolates (%)																	
Antimicrobial	0.002–0.75	1–1.5	2–3	4	6	8	12	16	24	32	48	64	96	128	256	Percentage of resistant isolates	MIC_50_	MIC_90_
Amikacin	-	-	-	-	3.4	-	3.4	3	7	6.9	10	6.9	3.4	-	55	55.2	256	256
Ampicillin	41.4	31	6.9	10	6.9	-	3.4	-	-	-	-	-	-	-	-	27.6	1	4
Cefepime	-	-	-	-	-	-	10	7	7	14	3.4	-	3.4	-	55	55.2	256	256
Cefoxitin	-	-	-	3.4	-	3.4	3.4	3	7	10	10	17	21	3.4	17	58.5	64	256
Ceftriaxone	-	-	-	3.4	3.4	17	17	7	7	10	-	6.9	10	3.4	14	34.5	24	256
Chloramphenicol	86.2	6.9	3.4	-	3.4	-	-	-	-	-	-	-	-	-	-	0	0.5	1
Ciprofloxacin	-	-	-	-	-	-	-	-	-	100	-	-	-	-	-	100	32	32
Gentamicin	3.4	6.9	21	31	21	10	-	-	-	3.4	-	-	-	-	3.4	3.4	4	8
Tetracycline	10.3	-	-	-	3.4	3.4	-	3	7	35	14	10	6.9	-	6.9	82.8	32	96
Sulfamethoxazole-trimethoprim	-	-	-	-	-	-	-	-	-	100	-	-	-	-	-	100	32	32

Distribution of the minimal inhibitory concentration (MIC) for the 29 isolates among the different antimicrobial concentrations (0.002–256 mg/µl). Shaded cells highlight the MIC range. Triple vertical bars mark the (approximate) breakpoint between sensitive and resistant. The resistance breakpoints are based on CLSI interpretative criteria for *Bacteroides fragilis* (ATCC 25285) (CLSI, 2012). No published MIC breakpoint for amikacin and gentamicin was found in the literature, therefore the highest concentration was considered as MIC.

MIC_50_, the MIC that inhibited at least 50% of the isolates; MIC_90_, the MIC that inhibited at least 90% of the isolates.

The multidrug resistance profile of the *Faecalibacterium sp.* isolates is shown in Table 2. A total of 19 different combinations of antibiotic resistance were observed. The isolates were resistant to, at least, 2 antibiotics (1 isolate) and up to 8 antibiotics (2 isolates). All 29 isolates tested showed multiresistance to ciprofloxacin and sulfamethoxazole-trimethoprim.

**Table 2:** Multidrug resistance profile of *Faecalibacterium prausnitzii* (n = 29 isolates) isolated from feces of dairy calves and piglets.

Resistance phenotype	Number of isolates
CIP-TRM	1
CIP-TRM-AMK	1
CIP-TRM-CEF	2
CIP-TRM-TET	1
CIP-TRM-AMK-TET	4
CIP-TRM-CEF-TET	2
CIP-TRM-CFX-TET	1
CIP-TRM-AMK-CEF-TET	1
CIP-TRM-AMK-CFX-TET	4
CIP-TRM-CEF-CET-TET	1
CIP-TRM-CFX-CET-TET	1
CIP-TRM-AMK-AMP-CEF-CFX	1
CIP-TRM-AMP-CEF-CET-TET	1
CIP-TRM-CEF-CET-CFX-TET	2
CIP-TRM-AMK-AMP-CEF-CET-TET	2
CIP-TRM-AMK-AMP-CEF-CFX-TET	1
CIP-TRM-AMK-AMP-CET-CFX-TET	1
CIP-TRM-AMK-AMP-CEF-CET-CFX-TET	1
CIP-TRM-AMK-AMP-CEF-CET-GET-TET	1

AMK, amikacin; AMP, ampicillin; CEF, cefepime; CET, ceftriaxone; CFX, cefoxitin; CIP, ciprofloxacin; GET, gentamicin; TET, tetracycline; TRM, sulfamethoxazole-trimethoprim.

### Phylogenetic analysis

**Figure 8:**
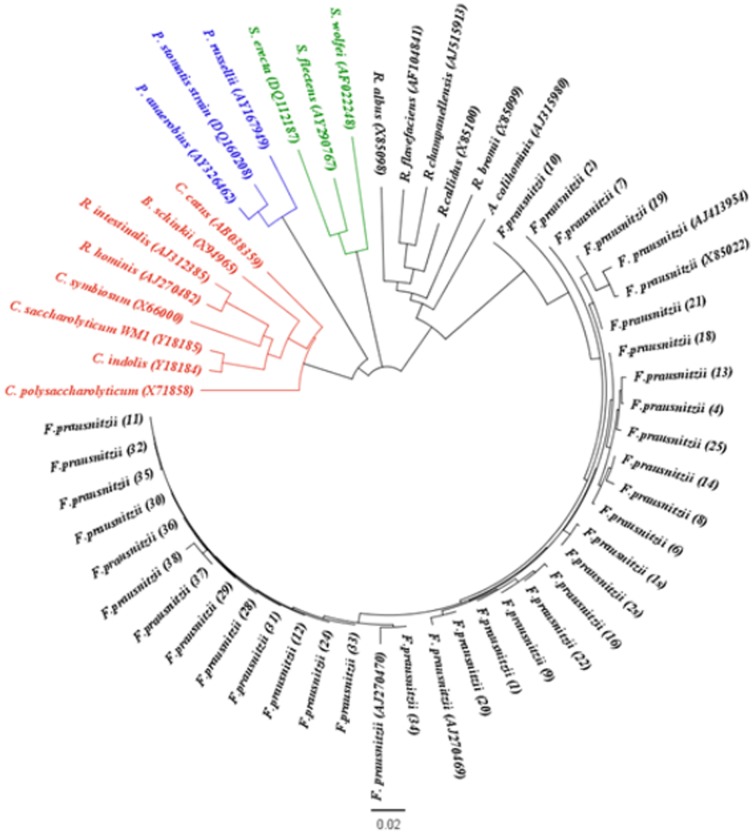
Phylogenetic tree showing the relationships of 16S rDNA sequences of 33 *Faecalibacterium prausnitzii* isolates from our group with other bacteria of the belonging to the same order; *Ruminococcaceae* (black), *Syntrophomonadaceae* (green), *Peptostreptococcaceae* (blue), *Clostridiaceae* and *Lachnospiraceae* (red).

A phylogenetic tree showing the relationships of 16S rDNA sequences from *Faecalibacterium* sp. with other bacteria belonging to the *Clostridiales* order and different families is shown in Fig. 8. The sequences from our isolates were closely related with other 4 *Faecalibacterium prausnitzii* sequences (AJ270469, AJ270470, AJ413954, X85022). Since our *Faecalibacterium sp.* sequences had 100% match with sequences from *Faecalibacterium prausnitzii*, and knowing that *prausnitzii* is the only specie described, we considered our isolates as *Faecalibacterium prausnitzii*.

## Discussion

Our study aimed the isolation and characterization of *Faecalibacterium prausnitzii* from feces of calves and piglets. *F. prausnitzii* has been the focus of many studies related to human health [5], [7], [9], and the anti-inflammatory mechanisms [8].

*F. prausnitzii* is extremely sensitive to oxygen and loses its viability if exposed to air for more than 2 minutes [10]. To survive in oxygenated niches within the human colon, *F. prausnitzii* uses an extracellular electron shuttle of flavins and thiols to transfer electrons to oxygen [30]. The media used by our group as a transport and growth media has cysteine and yeast extract, which provides thiol and flavin, respectively. In addition, the ruminal fluid supplies acetate and other micronutrients required for growth.

The measurement of the SCFA metabolized by our isolates indicated that *F. prausnitzii* is an acetate consumer and butyrate producer, as previously described [18], [31], [32]. However, a substantial variability in the butyrate production was observed among the isolates. In general, isolates that were high butyrate producers also demonstrated high *in vitro* growth performance (r = 0.68). The two isolates from piglets showed the best growth performance and produced higher amounts of butyrate than the bovine and the DSM 17677 isolates.

The growth in adverse conditions, such as low pH or the presence of bile salts was evaluated in order to mimic the conditions encountered by *F. prausnitzii in vivo*. The low pH in the stomach or in the ruminant's abomasum could prevent or reduce the growth of these bacteria. Our results are in agreement with previously reported pH values tolerated by *F. prausnitzii* [24].

The fasting gastric pH observed in humans is about pH 1.7, which rose and peaked at pH 6.7 postprandially, then declined gradually back to the fasted state value in the next 2 hours [33]. The same study measured the pH in the duodenum, which varied between pH 5.4 and 6.1. The successful use of *F. prausnitzii* as probiotic will most likely depend on the administration method. According to the results obtained in our study, oral administration of *F. prausnitzii* would be recommended after feeding to avoid the low gastric pH, which could be deleterious to the bacterial cells. *F. prausnitzii* isolates were susceptible to the presence of bile salts *in vitro*. It has been reported that patients with hepatic and intestinal disorders have increased luminal concentration of bile salts [34], which could explain the lower count of *F. prausnitzii* observed in those individuals.

On this study, all evaluated *F. prausnitzii* isolates were resistant to ciprofloxacin and sulfamethoxazole-trimethoprim. Additionally, most isolates were resistant to tetracycline, amikacin, cefepime and cefoxitin. To the best of our knowledge, this is the first study that evaluated antimicrobial susceptibility of *F. prausnitzii*. Resistance genes would allow the *F. prausnitzii* to survive and persist in the intestinal microbiota during antibiotic treatment; however, it could also act as reservoir of resistance genes and potentially transfer them to pathogenic bacterial species [35]. It is our future interest to study the molecular mechanisms for antibiotic resistance in *F. prausnitzii* as well its potential role in resistance dissemination. Furthermore, *F. prausnitzii* could be a used as a model bacterium for studies of antibiotic resistance, since it is one of the most abundant intestinal microorganisms.

Aminoglycosides' entry in the bacterial cell is most efficiently achieved by energy obtained from electron transport using oxygen (or, alternatively, nitrate) as a terminal electron acceptor [36]. Therefore, anaerobes are less susceptible to aminoglycosides than aerobic bacteria due to impermeability [37]. High levels of resistance to aminoglycoside were expected due to the anaerobic nature of the isolates; however, only 55.2% and 3.4% of our *F. prausnitzii* isolates were resistant to the highest concentrations of amikacin and gentamicin, respectively.

About 83% of the isolates were resistant to tetracycline (MIC ≥16 µg/ml), while the MIC of the same antibiotic was considerable lower (MIC <0.2 µg/ml) to the two swine and the human DSM 17677 isolates. The tetracycline resistance gene *tetW* is the most prevalent among anaerobic commensal gut bacteria and it has been found in the human fecal microbiota [38], [39], pig feces [40] and bovine rumen[41]. A possible tetracycline resistance gene could be present among isolates from calves. However, tetracycline is not an antibiotic routinely used by the farm where the calves' samples were collected. Resistance genes are present even in antibiotic-free animals and environmental sources such as water and soil are probably responsible for its dissemination [42].

In summary, our results suggested that the isolates were consuming acetate present in the media and producing butyrate (r = −0.87) and that butyrate production correlated positively with bacterial growth (r = 0.68). The optimal pH for growth ranged between 5.5 and 6.7, while most isolates were inhibited by of the lowest concentration of bile salts tested (0.1%). Antimicrobial resistance profile showed that most isolates of *F. prausnitzii* were resistant against ciprofloxacin and sulfamethoxazole-trimethoprim. More than 50% of the isolates were resistant to tetracycline, amikacin, cefepime and cefoxitin. A total of 19 different combinations of multidrug resistance were observed among the isolates. Our results provide new insights into the cultural and physiological characteristics of *Faecalibacterium prausnitzii* illustrating large variability in short chain fatty acid production, in vitro growth, sensitivity to bile salts, and antibiotic resistance and suggesting that future probiotic candidates should be carefully studied before elected for in vivo studies. The study of the use of the *Faecalibacterium prausnitzii* as a livestock probiotic is of our future interest, as well as the resistance genes and their potential transmission to relevant enteric pathogens such as *Salmonella* spp. and *E. coli*.
